# Approach–avoidance of facial affect is moderated by the presence of an observer-irrelevant trigger

**DOI:** 10.1007/s11031-016-9595-1

**Published:** 2016-11-02

**Authors:** S. B. Renard, P. J. de Jong, G. H. M. Pijnenborg

**Affiliations:** 1 Department of Clinical Psychology and Experimental Psychopathology, University of Groningen, Groningen, The Netherlands; 2 Department of Psychotic Disorders, GGZ Drenthe, Assen, The Netherlands

**Keywords:** Social cognition, Emotion, Context, Attribution, Static versus dynamic

## Abstract

This study examined whether approach–avoidance related behaviour elicited by facial affect is moderated by the presence of an observer-irrelevant trigger that may influence the observer’s attributions of the actor’s emotion. Participants were shown happy, disgusted, and neutral facial expressions. Half of these were presented with a plausible trigger of the expression (a drink). Approach–avoidance related behaviour was indexed explicitly through a questionnaire (measuring intentions) and implicitly through a manikin version of the affective Simon task (measuring automatic behavioural tendencies). In the absence of an observer-irrelevant trigger, participants expressed the intention to avoid disgusted and approach happy facial expressions. Participants also showed a stronger approach tendency towards happy than towards disgusted facial expressions. The presence of the observer-irrelevant trigger had a moderating effect, decreasing the intention to approach happy and to avoid disgusted expressions. The trigger had no moderating effect on the approach–avoidance tendencies. Thus the influence of an observer-irrelevant trigger appears to reflect more of a controlled than automatic process.

## Introduction

Facial affect is an important part of human communication, it signals the internal state of the actor and can elicit behavioural reactions in the observer (Horstmann 2003). While much research has focused on facial affect recognition in different cultures (e.g., Elfenbein and Ambady 2002), and problems with affect recognition in psychopathology (e.g., Renard et al. 2012), considerably less research has focused on the behavioural responses that are elicited by facial emotional expressions, and how these responses may depend on contextual cues. Insight in the possible context-dependency of behavioural response patterns is important for a more comprehensive understanding of the role of facial affect in daily interactions. Furthermore, differences in how contextual cues are used in responding to facial affect might play an important role in psychopathology, such as social anxiety.

The few studies that examined reactions towards facial affect focused on approach–avoidance related behaviour (Lang et al. 1997). Some of these examined controlled approach–avoidance intentions, which are measured explicitly, while others examined automatic approach–avoidance tendencies, which are measured implicitly. For example, happy expressions have been found to elicit approach tendencies (Seidel et al. 2010; Stins et al. 2011), and facial expressions of anger have been found to elicit avoidance tendencies (Heuer et al. 2007; Marsh et al. 2005; Seidel et al. 2010). Consistent with current dual process models that emphasize the relevance of differentiating between more impulsive and more reflective processes (e.g., Strack and Deutsch 2004), there is evidence that the more automatic approach–avoidance tendencies may diverge from self-reported intentions. For example, Seidel et al. (2010) showed that even though participants expressed the intention to avoid someone expressing sadness, they showed an automatic tendency to approach that person.

These findings are not merely the result of participants approaching positive stimuli (i.e., positive emotions) and avoiding negative stimuli (i.e., negative emotions). For example, someone expressing fear while looking in a certain direction elicits avoidance behaviour away from the apparent source of threat (Ozono et al. 2012). Thus, the expression of fear seems to be attributed to an observer-irrelevant threatening stimulus, which should be avoided. More generally, approach–avoidance related behaviour in response to facial affect might depend on what seems to trigger the emotional expression. In some cases that might be the observer, but in other cases it might be an observer-irrelevant stimulus. In line with this, Rozin et al. (1994) explain that a facial expression of disgust may be elicited by the observer (e.g., because of his or her appearance) or, for example, by something that the actor just consumed.

Thus far, no study directly examined the influence of a plausible, observer-irrelevant trigger of the expression on approach–avoidance related behaviour towards that facial expression, even though in daily interactions such triggers are often available and might have a large impact on behaviour. Therefore, the aim of the current study was to test how the presence of a plausible, observer-irrelevant trigger of the emotional expression might influence the observer’s approach–avoidance behaviour towards the actor. We specifically examined approach–avoidance related behaviour towards disgusted, happy, and neutral facial expressions in the absence or presence of a plausible trigger of the expression. These emotions were selected because they are relevant in social interactions (Adolphs 2002; Hutcherson and Gross 2011; Sherman and Haidt 2011) and could be triggered by the same observer-irrelevant stimulus (i.e., a drink). The presence of the drink may make it more intuitive for the observer to attribute the emotion to the taste of the drink than to him or herself. To test whether the observer-irrelevant trigger would influence approach–avoidance intentions and tendencies in the same way, both an explicit and an implicit measure of the observers’ approach–avoidance related behaviour were included in this study.

The following hypotheses were tested: (1) In the absence of an observer-irrelevant trigger, happy facial expressions elicit approach related behaviour, while expressions of disgust elicit avoidance related behaviour; and (2) the presence of a plausible, observer-irrelevant trigger has a moderating effect; decreasing approach related behaviour in response to happy facial expressions and avoidance related behaviour in response to expressions of disgust (i.e., there would be an interaction between the expressed emotion and the presence of the trigger).

The literature did not provide a solid basis to make specific predictions with regard to differences between more automatic approach–avoidance tendencies and more explicit intentions. We assessed both, as current dual process models emphasise the importance of differentiating between more impulsive (e.g., automatic approach–avoidance tendencies) and more reflective processes (e.g., explicit approach–avoidance intentions). No hypotheses were formulated with regard neutral expressions or to a main effect of the trigger. For exploratory purposes the experiment was conducted with both static and dynamic stimuli. The static stimuli allowed for maximal comparability with other research using implicit measures of approach–avoidance related behaviour while the dynamic stimuli more closely mimic real life expression (cf. Krumhuber et al. 2013; Recio et al. 2013). Therefore, similar but stronger effects were hypothesized for the dynamic stimuli.

## Methods

### Participants

Sixty-nine female participants were randomly recruited from a paid participant pool of the psychology department of the University of Groningen. The sample consisted of women to make the experiment more sensitive, as women show a higher sensitivity towards disgust than men (Curtis et al. 2004; Haidt et al. 1994). The mean age of the sample was 24.4 years (*SD* = 6.7). Sixty-five participants were graduate or undergraduate students from the University of Groningen, three participants had finished their undergraduate degree and one participant had finished her graduate degree. The sample size gave a power >.80 to find medium to large effects (Cohen 1988).

### Materials

#### Stimuli

**Fig. 1:**
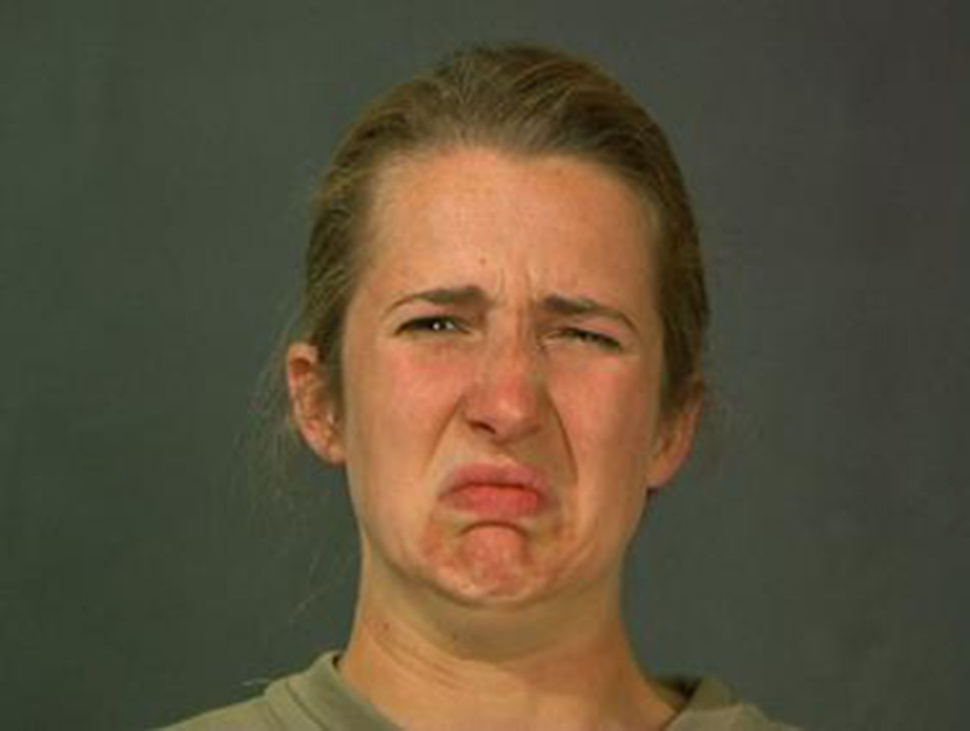
Disgust expressed without a trigger

**Fig. 2:**
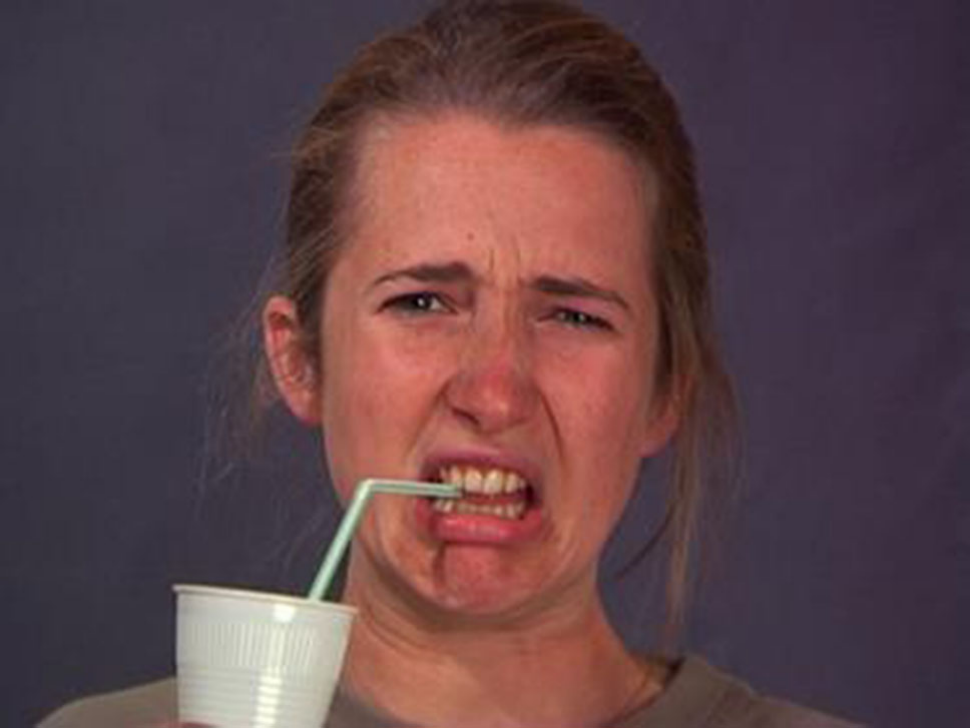
Disgust expressed with a trigger

A combination of dynamic and static stimuli was used. The dynamic stimuli were 3 s video-clips of three male and three female actors against a grey background directly looking at the camera. The expressions of these actors started neutral and either stayed neutral or changed into an expression of disgust or happiness. All actors displayed each emotion twice, once without any trigger and once while having a drink, resulting in 36 dynamic stimuli (Jabbi et al. 2007; van der Gaag et al. 2007). The 36 static stimuli were snapshots of the last frame of the video-clips, at which point the expressed emotion was clearly visible (see Figs. 1, 2). All stimuli were 768 px wide and 576 px high.

#### Explicit measure

The explicit intention to approach or avoid an expression was assessed through a questionnaire. The questionnaire contained two questions per stimulus, translated from Dutch these questions were: (1) “What emotion do you recognize?” and (2) “To what extent would you approach or avoid this person?” The first question was a multiple choice question with happiness, disgust, or no emotion as response options and was used to assess the quality of the stimuli. The second question assessed the explicit intention to approach/avoid the stimuli and was answered on a visual analogue scale (VAS-scale) ranging from 0 (“definitely avoid”) to 100 (“definitely approach”). The static and dynamic stimuli were presented mixed and in a completely random order. Participants were not directly asked to what extent they attributed the emotion to themselves or to the drink to keep the actual aim of the study hidden for the participants.

#### Implicit measure

The automatic tendency to approach or avoid a stimulus was assessed with the Manikin version of the affective Simon test (AST_manikin; De Houwer et al. 2001), because this test has proven to be a sensitive and powerful measure of approach–avoidance tendencies (Krieglmeyer and Deutsch 2010).

The AST_manikin is a reaction time computer task, in which participants have to move a manikin as quickly as possible towards or away from a stimulus using the arrow keys. The manikin appeared randomly above or below the stimulus to prevent participants from recoding the required responses into anything other than “towards or away from the stimulus”. Participants had to move the manikin depending on the colour balance of the stimulus. Thus, the facial expression and presence or absence of the trigger were task-irrelevant features. Each stimulus was presented once as 25 % more green than the original and once as 25 % more blue than the original and as a result each stimulus had to be avoided once and approached once. In addition to the stimuli described earlier eight checkerboard patterns were added as truly neutral stimuli, four were 25 % more blue and four were 25 % more green. All stimuli were presented in completely random order with an inter-stimulus-interval of 1 s. A new stimulus was only presented after the manikin was moved far enough in the correct direction (five steps from the starting position of the manikin). The test was counterbalanced to control for the possible effect of the colour balance.

We used two versions of the AST_manikin: a standard version with the static stimuli and an adapted version with the dynamic stimuli. Both versions started with four practice trials. The standard version contained 80 experimental trials (72 facial stimuli + 8 control stimuli). In addition to the dynamic stimuli, the adapted version of the AST_manikin contained static stimuli for which the task relevant features (i.e., colour balance and manikin) appeared after 3 s (3 s delay stimuli). These stimuli were added because the task relevant features also appeared after 3 s in the dynamic stimuli (i.e., in the last frame). We wanted to control for the possible effect of this delay as it is absent in the standard AST_manikin. This resulted in the adapted AST_manikin having 152 experimental trials (72 dynamic + 72 3 s delay + 8 control stimuli).

### Procedure

All procedures were approved by the institutional review board of the Heymans Institute of the University of Groningen. Participants gave written informed consent. Following the recommendation of Bosson et al. (2000) participants started with the implicit tasks as this minimizes the influence of carry-over effects. Participants first completed the standard version of the AST_manikin task and continued with the adapted version. Participants were instructed to move the manikin, as fast and accurate as possible, towards or away from the stimulus depending on its colour. They were further instructed that the colour of the stimuli was unrelated to its content and that the task would not continue unless they moved the manikin far enough in the correct direction. After a short break, participants completed the questionnaire measuring the explicit intention to approach or avoid the stimuli.

### Analyses

Examining the reaction times on the AST_manikin of moving towards or away from the stimuli independent of each other is uninformative as participants might both be faster in approaching and avoiding certain stimuli than other stimuli. In line with previous research using this type of approach–avoidance tasks (e.g., Heuer et al. 2007), we therefore relied on the relative tendency to approach or avoid the target stimulus, which was computed by subtracting the reaction time of moving the manikin towards a stimulus from the reaction time of moving the manikin away from the same stimulus. Similar to the procedure used by Krieglmeyer and Deutsch (2010), reaction times were only used if the initial response was correct and if it occurred between 150 and 1500 ms after task relevant stimulus features were presented. Faster reactions are thought to be random responses and slower reactions are thought to reflect the participant having a lapse in concentration. Originally, we intended to compare the approach–avoidance tendencies with the neutral checkerboard pattern. However, participants had difficulty distinguishing the blue and green checkerboard stimuli from each other resulting in mostly random responses to these stimuli. Therefore, the approach–avoidance tendencies can only be interpreted relative to each other, where a higher score reflects a relatively stronger tendency to approach a stimulus and a lower score a relatively stronger tendency to avoid it.

The data were analysed with SPSS version 21.0. The study has a within subjects design with the explicit and implicit measure of approach–avoidance behaviour as dependent variables. The following stimulus characteristics were used as within-subject independent variables: emotion (happy, disgust, no emotion), trigger (no trigger, drink as trigger), and presentation (static, dynamic, and 3 s delay). The main analyses consisted of two sets of repeated measures analyses of variance (RM-ANOVAs). The first set of analyses focused on the static stimuli, the second set of analyses also examined the dynamic stimuli. Each set contained one analysis for the explicit measure and one for the implicit measure of approach–avoidance behaviour.

## Results

The emotions were recognized correctly 97 % of the time (*SD* = 5.81). Following *t* tests showed that this was not influenced by the emotion, the gender of the actor, the availability of a trigger or whether the stimulus was static or dynamic.

### Static stimuli

The three emotion (happy, disgust, neutral) × 2 trigger (no trigger, drink trigger) RM-ANOVA on the explicit measure showed a main effect of emotion, *F*(2,67) = 291.07, *p* < .001, η^2^ = .49, and trigger availability, *F*(1,68) = 15.03, *p* < .001, η^2^ = .18. Most important for the current study, the interaction of emotion × trigger was also significant, *F*(2,67) = 55.24, *p* < 0.001, η^2^ = .87.

**Fig. 3:**
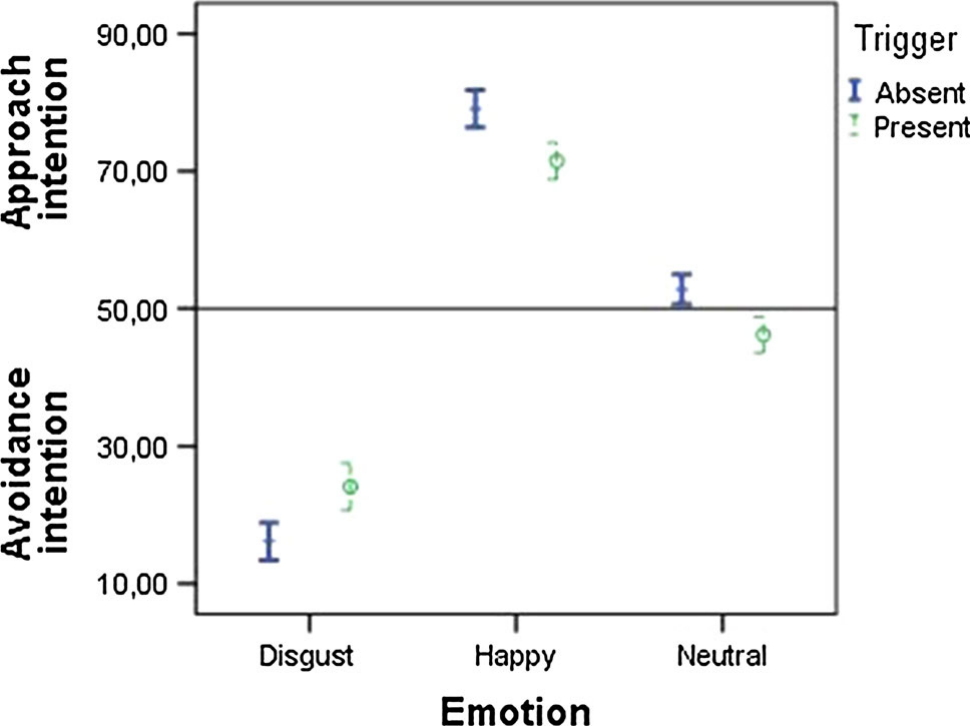
Approach–avoidance intention (mean + 95 % CI) in response to the actor expressing disgust, happiness or a neutral expression for static stimuli in the absence or presence of the trigger

The interaction effect showed that the strength of the intention to approach or avoid someone expressing a specific emotion depended on the presence of a plausible, observer-irrelevant trigger (see Fig. 3). Participants showed a significantly stronger, *t*(68) = −6.15, *p* < .001, *d* = 0.76, intention to avoid someone expressing disgust when the trigger was absent, 95 % CI = [13.42, 18.87] than when the trigger was present, 95 % CI = [20.69, 27.54]. Participants showed a significantly stronger, *t*(68) = 8.72, *p* < .001, *d* = 1.05, intention to approach someone expressing happiness when the trigger was absent, 95 % CI = [76.36, 81.79] than when it was present, 95 % CI = [68.82, 74.18]. Individuals reported an intention to approach someone with a neutral expression when the trigger was absent, 95 % CI = [50.60, 54.95], but to avoid that person when the trigger was present 95 % CI = [43.56, 48.82]. This difference was significant, *t*(68) = 7.48, *p* < .001, *d* = 0.93. These results support the hypothesis that approach–avoidance related behaviour towards facial expressions is moderated by the presence of a plausible, observer-irrelevant trigger of those expressions.

**Fig. 4:**
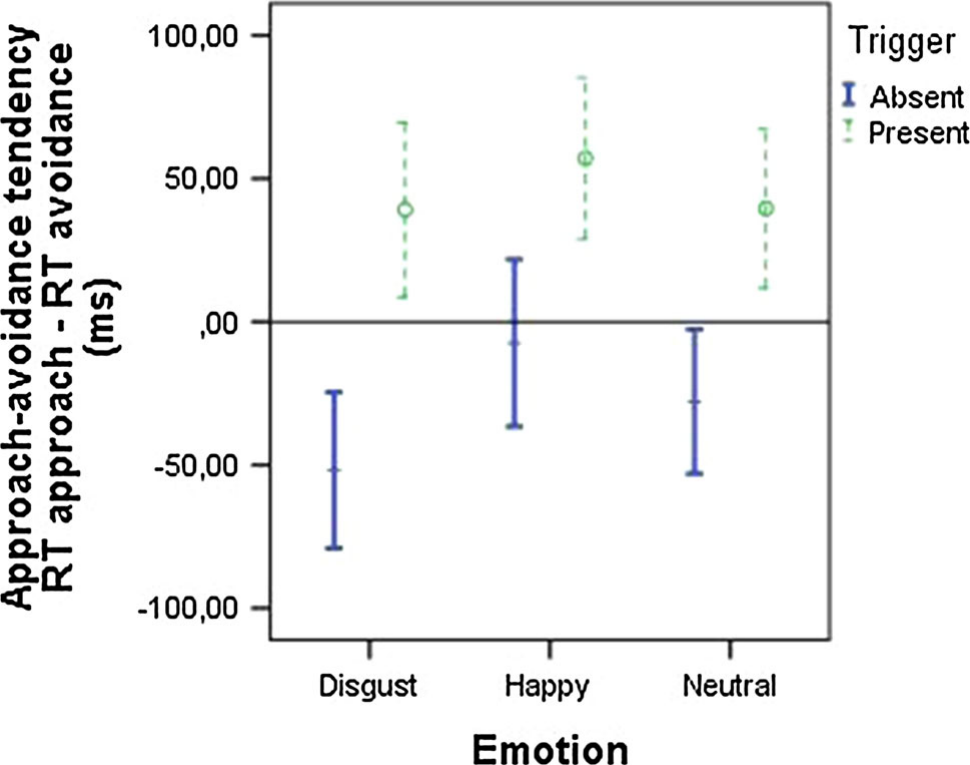
Approach–avoidance tendency (mean + 95 % CI) in response to the actor expressing disgust, happiness, or a neutral expression for static stimuli in the absence or presence of the drink

The three emotion (happy, disgust, no emotion) × 2 trigger (no trigger, drink trigger) RM-ANOVA on the AST_manikin showed a significant main effect of emotion, *F*(2,67) = 3.18, *p* = .048, and trigger availability, *F*(1,68) = 22.50, *p* < .001, on the tendency to approach or avoid the stimuli. However, unlike with the explicit measure, there was no interaction between trigger and emotion, *F*(2,67) = 0.64, *p* = .53. There was a significantly stronger avoidance tendency toward expressions of disgust than towards happy expressions, *t*(68) = 2.54, *p* = 0.013, *d* = 0.36. The results for neutral expressions did not differ significantly from happy, t(68) = 1.11, *p* = 0.27, or disgust expressions, t(68) = −1.55, *p* = 0.13. Figure 4 shows that the availability of a plausible, observer-irrelevant trigger reduced the relative avoidance tendency for all emotions. Follow-up paired *t* tests showed a significant difference between expressions with and without a trigger for disgust, *t*(68) = −4.02, *p* < .001, *d* = 0.48, happy, *t*(68) = 2.80, *p* = .007, *d* = 0.34, and neutral expressions, *t*(68) = 3.53, *p* = .001, *d* = 0.43.

### Dynamic stimuli

**Fig. 5:**
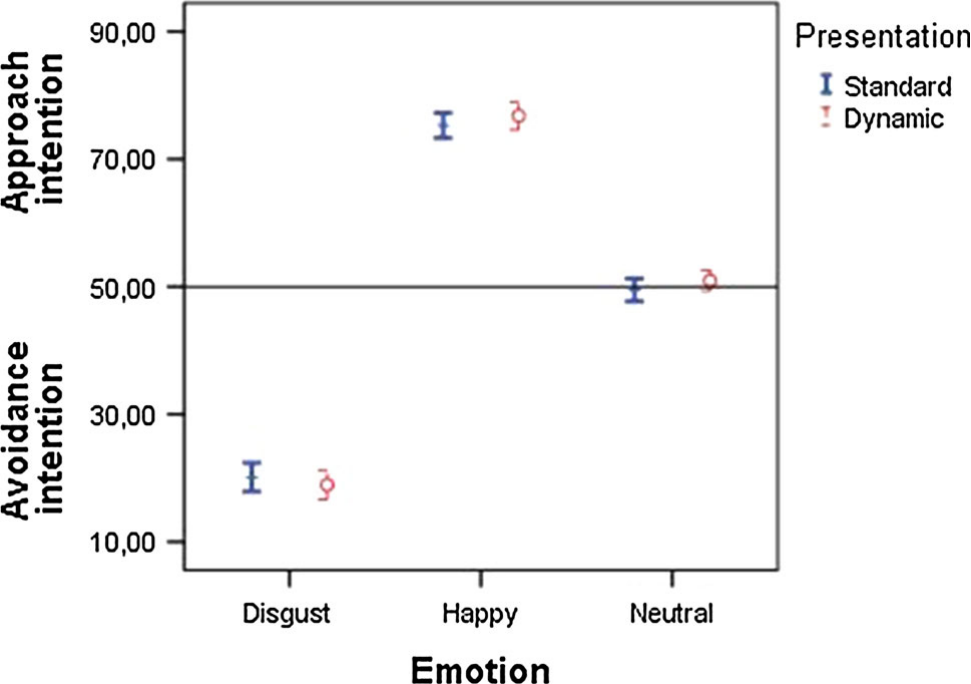
Approach–avoidance intention (mean + 95 % CI) in response to the actor expressing disgust, happiness, or a neutral expression for static and dynamic stimuli

The second set of analyses included the dynamic stimuli (in comparison with the static stimuli). The three emotion (happy, disgust, neutral) × 2 trigger (no trigger, drink trigger) × 2 presentation (static, dynamic) RM-ANOVA on the explicit measure showed a main effect of emotion, *F*(2,67) = 304.30*, p* < .001, a main effect of trigger, *F*(1,68) = 15.14, *p* < .001, and an interaction effect between emotion and trigger, *F*(2,67) = 55.86, *p* < .001. These results are similar to what was found when only looking at the static stimuli, as evidenced by the absence of an emotion × trigger × presentation interaction, *F*(2,67) = 1.61, *p* = .208 (see also Fig. 5). There was no significant main effect of presentation, *F*(1,68) = 3.74*, p* = .057, but there was a significant interaction between presentation and emotion, *F*(2,67) = 10.81*, p* < .001. Post-hoc analyses showed that the intention to avoid someone expressing disgust and approach someone expressing happiness was stronger when the expressions were presented as video-clips than when they were presented as pictures, *t*(68) = −2.51, *p* = .014, *d* = 0.30, and *t*(68) = 3.06, *p* = .003, *d* = 0.39, respectively. Compared to pictures, the intention to approach someone with a neutral expression was also stronger when the stimuli were presented as video-clips, *t*(68) = 2.89, *p* = .005, *d* = 0.35.

**Fig. 6:**
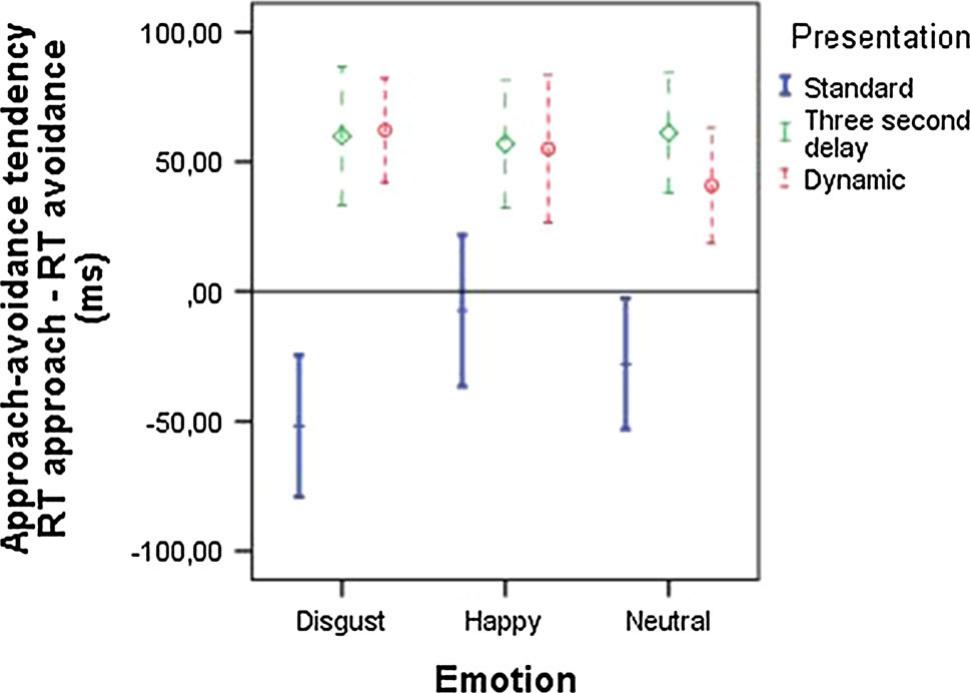
Approach–avoidance tendency (mean + 95 % CI) in response to the actor expressing disgust, happiness or a neutral expression for static, 3 s delay, and dynamic stimuli

Figure 6 shows that the effects found in the standard Manikin task on the automatic tendency to approach or avoid static stimuli disappeared when looking at the dynamic stimuli and at the 3 s delay stimuli in the adapted Manikin task. A three emotion (happy, disgust, neutral) × 2 trigger (no trigger, drink trigger) × 3 presentation (static, dynamic, 3 s delay) RM-ANOVA only showed a main effect of presentation, *F*(2,67) = 27.95*, p* < .001. Unexpectedly, there was no effect of emotion, *F*(2,67) = 0.61*, p* = .547, nor was there an interaction between emotion and trigger, *F*(4,65) = 1.80*, p* = .14. As can be seen in Fig. 6, the main effect of presentation indicates that participants overall showed stronger approach tendencies to the dynamic and 3 s delay stimuli than to the static stimuli, *t*(68) = −6.22, *p* < .001, *d* = 0.75, and *t*(68) = −7.06, *p* < .001, *d* = 0.85, respectively, while no difference was seen between the dynamic and 3 s delay stimuli, *t*(68) = −0.92, *p* = .36.

## Discussion

In line with our hypotheses, the results of this study indicate that individuals’ explicit intention to approach someone expressing happiness, and their intention to avoid someone expressing disgust are attenuated when a plausible observer-irrelevant trigger is added to the facial stimuli. Specifically, people expressed a weaker intention to approach someone with a happy expression, when that expression could be triggered by something other than the observer. In contrast, people expressed a weaker intention to avoid someone expressing disgust when it could be triggered by something other than the observer. However, the relatively stronger avoidance tendency towards expressions of disgust than towards expressions of happiness was not attenuated by the availability of the trigger, instead the availability of the trigger increased the approach tendency irrespective of the emotion that was expressed. As the current results show that the trigger moderated the observer’s conscious intention to approach or avoid the actor but not their automatic approach–avoidance tendencies, it seems that the effect of the trigger requires some sort of active processing and does not reflect a mostly automatic (pre-attentive) process.

Aside from showing that intentional approach–avoidance behaviour is influenced by the presence of a plausible trigger, this study contributes to our understanding of the reactions that are elicited by facial expressions of disgust and happiness. Consistent with previous research (Seidel et al. 2010; Stins et al. 2011), individuals reported the intention to approach someone with a happy expression and to avoid someone who expressed disgust. Similarly, there was a stronger implicit approach tendency towards someone with a happy expression than towards someone with a disgusted expression. Although not directly relevant for our research questions, the results of the implicit task additionally indicated that adding a drink as an observer-irrelevant trigger of the actor’s emotion generally enhanced the automatic approach tendencies. This was unexpected but is in line with an earlier study showing that food stimuli in general elicit automatic approach behaviour (Machulska et al. 2015).

The explicit intention to approach or avoid dynamic stimuli was similar in direction but stronger than the intention to approach or avoid static stimuli. In addition, the effect of the observer-irrelevant trigger was similar for static and dynamic stimuli. This was, however, not found for the automatic tendency to approach or avoid dynamic stimuli. The automatic approach and avoidance tendencies that were found for static stimuli appear to be limited to a short time span; after 3 s this automatic tendency completely disappeared as shown by the dynamic and 3 s delay stimuli. This can be explained by the emotions no longer influencing the automatic reactions because the participants were able to process the emotions before the task relevant features were shown. As a result, participants could focus on the task relevant features (colour and position of the manikin) and the emotion no longer influenced task performance. In other words, by using video-clips the AST_manikin no longer seemed to index automatic approach–avoidance tendencies. This is important for future research as these findings seem to indicate that the sensitivity of the AST as an index of automatic tendencies is limited to a short time span.

Although the sample was restricted to women, mostly in their young adulthood with above average intelligence, we do not expect the results to be limited to this population. Based on earlier research (Bradley et al. 2001), men are expected to show similar but smaller effects than women in such an experiment. Intelligence is not expected to have any effect because the tasks in this study were not cognitively intensive. Additional research is needed to rule out an effect of age, but we don’t expect age to have an effect on these basic processes other than an overall slowing on the AST_manikin.

Future research should investigate whether these results generalize to other emotions and other triggers. For example, are expressions of anger also avoided less when there is a plausible observer-irrelevant trigger of the actor’s emotional expression? It is also important to examine whether the results of this study generalize to actual approach or avoidance behaviour. This could be done by using a task similar to Stins et al. (2011), who measured approach and avoidance tendencies through whole body forward or backward movements, or even better through real time interactions.

To conclude, the present study showed that both the observers’ intentions and their more automatic tendencies to approach or avoid particular faces varied as a function of the actor’s emotional expression. The present study was the first to show that the impact of the emotional expression on the observer’s (explicit) intention to approach or avoid the actor was moderated by the absence or presence of a plausible observer-irrelevant trigger of the emotion. The finding that the influence of the trigger was restricted to the explicit measure seems to imply that the influence of the trigger requires some processing and does not reflect a merely reflexive response. From a clinical perspective it would be important for future research to examine whether the availability of such a potential trigger of a particular emotional expression has differential effects in certain clinical populations. For example, it would be interesting to explore whether patients with paranoid delusions or social phobia might tend to ignore the potential relevance of contextual triggers and persist in overestimating their own role as a trigger of other people’s (negative) emotional expressions.
